# Frege and the origins of model theory in nineteenth century geometry

**DOI:** 10.1007/s11229-019-02421-4

**Published:** 2019-10-10

**Authors:** Günther Eder

**Affiliations:** Department of Philosophy, University of Vienna, Universitätsstrasse 7, 1010 Vienna, Austria

**Keywords:** Frege, Early metatheory, History of model theory, Nineteenth century geometry

## Abstract

The aim of this article is to contribute to a better understanding of Frege’s views on semantics and metatheory by looking at his take on several themes in nineteenth century geometry that were significant for the development of modern model-theoretic semantics. I will focus on three issues in which a central semantic idea, the idea of reinterpreting non-logical terms, gradually came to play a substantial role: the introduction of elements at infinity in projective geometry; the study of transfer principles, especially the principle of duality; and the use of counterexamples in independence arguments. Based on a discussion of these issues and how nineteenth century geometers reflected about them, I will then look into Frege’s take on these matters. I conclude with a discussion of Frege’s views and what they entail for the debate about his stance towards semantics and metatheory more generally.

## Introduction

Gottlob Frege was arguably the first to establish a formal system of logic in an essentially modern sense. In the course of justifying his logicist thesis, he created a formal language to represent propositions in a perspicuous way and devised deductive systems that enabled him to represent gap-free derivations. From a contemporary point of view though, Frege’s presentations also contain some striking lacunae. Frege never precisely defined a semantics for his formal systems in the now-standard way, he never defined a relation of logical consequence in terms of this semantics, and he never explicitly raised the question of whether his systems are sound and complete with respect to this consequence relation in a way we’ve grown accustomed to.

According to an influential tradition in Frege-scholarship, tracing back to Van Heijenoort (1967), these omissions are no coincidence. According to this tradition, they are expressions of a fundamental trait in Frege’s conception of logic, his *universalism*, which prevented him from stepping outside his systems and raising metalogical questions, specifically questions relating to semantics. Tom Ricketts, for example, claims that “anything like formal semantics, as it has come to be understood in light of Tarski’s work on truth, is utterly foreign to Frege” (Ricketts 1986, p. 67). In particular, according to this interpretive tradition, Frege was unable to make sense of all forms of reasoning that involve the idea of ‘reinterpreting’ non-logical terms.^1^ James Conant notes that “the distinction between a formal system and its interpretation is entirely alien to the Begriffsschrift” and that “questions concerning [a sign’s] disinterpretation or reinterpretation do not arise” (Conant 1992, p. 171, en.  58). Jaakko Hintikka, even more drastically, asserts that “[for Frege] model theory was impossible” (Hintikka 1988, p. 1).

Now, there is a serious danger of anachronism when it comes to claims to the effect that certain ways of reasoning that we would now classify as ‘semantic’ or ‘model-theoretic’ were alien or familiar to Frege. After all, semantics as we know it, and model-theoretic semantics in particular, are of relatively recent origin. Still, we may legitimately ask ourselves what Frege thought about issues that were closely connected to the gradual development of model-theoretic thinking. In order to restrict ourselves to a manageable task, I want to focus on three salient issues in nineteenth century geometry that are relevant: the introduction of elements at infinity in projective geometry; the idea of transfer principles, especially the principle of duality; and the use of counterexamples in independence arguments. Each of these topics has been discussed in the secondary literature on Frege in one form or another by people like Patricia Blanchette, Tom Ricketts, Jamie Tappenden, Mark Wilson, Michael Hallett, Paolo Mancosu, and others. The general aim of this article is to gain a better understanding of Frege’s views by bringing together and following up on these discussions.

The plan for this article is as follows: In the next section I will try to identify more precisely what we will be looking for, namely the idea that parts of mathematical language can be *reinterpreted*. The third section will be concerned with tracing this idea in nineteenth century geometry by focussing on the aforementioned themes: the introduction of elements at infinity, the principle of duality, and the use of counterexamples in independence proofs. In the fourth section, I will discuss how Frege reflected about these issues, concluding that Frege indeed rejects the notion of mathematical language as being reinterpretable. The last section will be concerned with a discussion of why this is so and what this might teach us about the controversy over Frege’s views on semantics and metatheory.

## The notion of a reinterpretable language

Contemporary model-theoretic semantics starts off with *formal languages*, typically *first-order* languages. A first-order language $L({\sigma })$ is determined by a set of *logical constants*, i.e.  some fixed set of truth-functional connectives and quantifiers, and a set of *non-logical constants* $\sigma$ that consists of individual constants, and relation- and function symbols of some specified arity. Formulas of $L(\sigma )$ can be formed by concatenating primitive terms according to certain formation rules. The central notion of model theory, then, is that of an $L({\sigma })$-*structure*, which is determined by a non-empty set *D* that specifies a domain for the quantifiers, and an interpretation function *I* that assigns interpretations to each non-logical constant. Each constant symbol is assigned some individual from the domain and relation- and function symbols are assigned relations and functions over the given domain according to their arity. Given some range of $L({\sigma })$-structures, we can then inductively define the relation of an $L(\sigma )$-*formula being true in an* $L(\sigma )$-*structure* in a sufficiently rich metalanguage. Given some language $L({\sigma })$, model theory then studies theories of this language by investigating the $L({\sigma })$-structures in which they are true or false.

It is important to note that this textbook build-up of model theory is of relatively recent origin and several things had to fall into place first. The list of ingredients includes, first and foremost, the notion of a formal language. With that comes an understanding of the distinctions between syntax and semantics and between object- and metalanguage as well as the specific distinction between logical and non-logical constants. Model theory then relies on the idea that the logical terms of a formal language have a fixed interpretation, while non-logical terms can be assigned different interpretations relative to some structure. Finally, model theory is based on the notion of a sentence being true in a structure, and that truth in a structure can be defined recursively in a sufficiently rich (typically informal set-theoretical) metalanguage.^2^

All of the aforementioned ingredients are important for contemporary model theory as a mathematical discipline. However, in this article I will only be concerned with the informal notion that seems to be prerequisite for all of this, the idea that a language, or parts of it, can be *reinterpreted*. What I mean by that is the informal idea that we can think of the interpretation (meaning, content, etc.) associated with certain terms as somehow *variable*, that the interpretation (meaning, content, etc.) commonly associated with an expression is not *glued* to it, and that, furthermore, sentences can be evaluated with respect to different interpretations associated with certain terms. For this notion to make sense, several things have to be in place. First, the notion of reinterpretation that we’ll be interested in only makes sense for a reasonably delineated part of mathematical discourse with specific primitive terms. Secondly, it requires a reasonably clear separation of a sign and what it designates. And, thirdly, it presupposes a distinction (if only implicit) between logical and non-logical terms. Of course, all of these requirements are to some extent still vague. As a consequence, the idea of mathematical language as being reinterpretable is vague itself and leaves room for discussion about particular cases. As we will see though, it will serve our purposes.

The notion of mathematical sentences as reinterpretable schemas has its roots in several different branches of mathematics. Since tracing this notion in all its facets is beyond the scope of this article, I will confine myself to discussing three specific issues in nineteenth century geometry that seem to be significant pieces of a complete story. I will hint at other issues throughout the article, though mostly in footnotes.

## Reinterpretable languages and nineteenth century geometry

The development of mathematics in the nineteenth century, and geometry in particular, is marked by a number of innovations and fundamental shifts.^3^ At the turn of the nineteenth century, Euclid’s *Elements* had been around for more than 2000 years and several shortcomings had become apparent by that time. Like nineteenth century mathematicians in general, geometers had acquired a new sense of rigour and voices became louder that were calling for new foundations for geometry. Towards the end of the nineteenth century, prominent figures like Moritz Pasch, David Hilbert, Guiseppe Peano, and others, were working on providing such foundations in the form of rigorous axiom systems for geometry. These efforts went hand in hand with two important trends. The first was the rise of projective geometry in the first half of the nineteenth century, and the other was the development of non-Euclidean geometry. We’ll get back to the latter later on, but for now we’ll focus on projective geometry.

Projective geometry has its roots in the study of perspective during the renaissance, when painters, architects and mathematicians became interested in problems relating to central projections. Projective geometry differs from traditional, Euclidean geometry in two respects. First, unlike Euclidean geometry, projective geometry is in the first instance only concerned with geometrical properties that relate to the *positions* of points, lines and planes relative to each other. Thus, metrical notions like distance, length, area, angle etc. are ruled out. Secondly, projective geometry differs from traditional geometry in that the notion of *parallelism* is ruled out. Instead, parallel lines are said to ‘meet’ at some ‘point at infinity’ and all these ‘points at infinity’ lie on a ‘line at infinity’. (In solid geometry, there are infinitely many ‘lines at infinity’, one corresponding to each set of parallel planes, and, in addition, there is a ‘plane at infinity’ on which all of these ‘lines’ lie.) It is these ‘elements at infinity’ that will be our first topic.^4^

### Elements at infinity

As indicated, the introduction of elements at infinity originated in the study of perspective, the idea being that, from a particular viewpoint, parallel lines meet at a vanishing point which lies at the horizon line. But elements at infinity soon developed a life of their own because of their usefulness in pure mathematics, and it was this usefulness that made them attractive for nineteenth century geometers. To illustrate, consider the following proposition:

#### Theorem 1

**Fig. 1:**
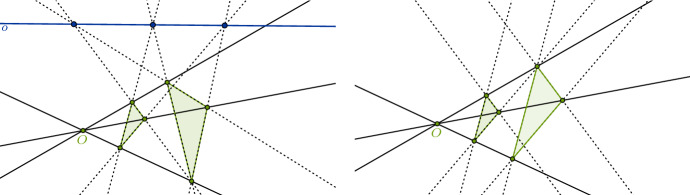
Desargues configurations with non-parallel sides (left) and with parallel sides (right)

If the lines joining corresponding vertices of two triangles meet in a point *O*, then the intersection points of corresponding sides of the triangles lie on a line *o*. (See Fig. 1, left.)

Theorem 1 is a plane incidence theorem, that is, its statement only mentions *points*, *lines* and the relation of a point *lying on* a line (incidence), and it is indeed a *theorem* about the Euclidean plane *if* we further assume that the intersection points of corresponding sides of the triangles *exist*. However, if these intersection points do not exist because corresponding pairs of sides are parallel, then the theorem doesn’t apply. Still, in this case we have another theorem:

#### Theorem 1*

If the lines joining corresponding vertices of two triangles meet in a point *O*, then, if two pairs of corresponding sides are parallel, then the third pair of sides is parallel. (See Fig. 1, right.)

Obviously, the two theorems are very similar. After all, they have the same antecedent. So one might hope to find a more general theorem of which both are instances. Indeed, such a general theorem, called *Desargues’ Theorem*, is precisely what we get if we interpret Theorem 1 in such a way that the term “point” not only refers to ordinary points but also points at infinity and the term “line” not only refers to ordinary lines but also some line at infinity. On this understanding, the original, Euclidean reading of Theorem 1 is covered. But, in addition, Theorem 1$^*$ is now covered as a special case as well. It’s just that in this case the intersection points are points at infinity and the line where they meet is the line at infinity.^5^

As this simple example illustrates, the introduction of elements at infinity has the potential to greatly facilitate the systematization of geometry, and all that seems to be required is a minor shift in terminology. If we designate what is common to parallel lines as a ‘point at infinity’ and extend the range of applicability of the term “point” to include these new elements (similarly for lines and planes), then a variety of geometrical propositions receive a uniform expression. Indeed, that’s precisely the advantage that was typically emphasized by nineteenth century geometers. It is because of this greater generality (among other things) that by the middle of the nineteenth century projective geometry had been widely regarded as the basis for all of geometry.^6^

The idea that the transition from ordinary points to the more general notion is effected by a mere change of terminology, introduced to achieve greater generality and simplicity, was widely shared by geometers in nineteenth century. But it raises some questions. Is talk about points at infinity a mere *façon de parler*, or does it have some kind of ontological import? How can we be sure that this way of speaking doesn’t involve any contradictions?^7^ What follows from this terminological shift for our conception of mathematical language? For example, does the sentence “Each pair of lines determines a unique intersection point” express a unique proposition or do we have to live with the fact that some mathematical sentences do *not* express unique propositions?

Different proposals have been made to put projective geometry and its use of elements at infinity on a solid footing. For example, Theodor Reye notes in his widely used textbook that “the statements ‘parallel lines have the same direction’ and ‘they contain the same infinitely distant point’ mean the very same thing [bedeuten das Nämliche]” (Reye 1866, p. 16), which seems to indicate that talk about elements at infinity may somehow be reduced to talk about ordinary lines and their directions. Similar proposals that rely on some kind of ‘abstraction’ have been made throughout the nineteenth century.^8^ Sometime around the Thirties of the nineteenth century, Julius Plücker and others had also introduced homogeneous coordinates, which enabled the study of projective geometry (including elements at infinity) by analytic means similar to those used in Euclidean geometry.^9^ Still, the foundations of projective geometry and the treatment of elements at infinity remained a matter of contention throughout the nineteenth century.

With hindsight, it might seem obvious to conceptualize the transition from Euclidean to projective geometry in terms of reinterpretable languages. On such a view, words like “point” and “line” are schematic terms that are open to reinterpretation and a sentence like “Every pair of lines determines a unique intersection point” may be true with respect to some interpretations, but false with respect to others. In particular, it is false when evaluated with respect to (some model of) the Euclidean plane, but true when evaluated with respect to (some model of), say, the extended Euclidean plane. It is hard to tell who was the first to articulate such a view explicitly in the context of projective geometry in connection with elements at infinity. Traces of such a view can be found already in Moritz Pasch’s famous *Vorlesungen über Neuere Geometrie* from 1882.^10^ Still, it took some time before this point of view found its way into mainstream mathematics. It was certainly accepted though around the turn of the twentieth century, at a time when a number of geometers had been working on axiom systems for both Euclidean and projective geometry, including people like Hilbert in Germany or Peano, Pieri, and Fano in Italy. One clear-cut example is Veblen and Young’s *Projective Geometry* (Veblen and Young 1910). In their axiomatic treatment of projective geometry, words like “point” or “line” are no longer tied to a particular interpretation, and sentences that contain these words are no longer tied to a particular truth value. Indeed, according to Veblen and Young, geometrical terms “are to be regarded as mere symbols devoid of content, except as implied by the fundamental propositions” (Veblen and Young 1910, p. 1). And while Veblen and Young use a particular “concrete representation” in terms of numerical coordinates to establish the consistency of the axioms of projective geometry, others are not ruled out. Since in this analytic representation all ‘points’ are on equal terms, there really is no further mystery about ‘points at infinity’.

### The principle of duality

The second issue I want to discuss in connection with the emergence of the concept of a reinterpretable language is the principle of duality in projective geometry. Actually, there are several duality principles, all of them instances of an even more encompassing class of principles that are sometimes referred to as *transfer principles*, i.e.  principles that state a systematic correlation of truths about one domain of objects with truths about some other domain of objects. Duality principles had been discovered sometime around the turn of the nineteenth century. The simplest version states that for every theorem in plane projective geometry we get another theorem by simply interchanging the terms “point” and “line” and, accordingly, the basic relation of a point lying on a line by the relation of a line passing through a point.^11^ To see duality in action, let’s look again at Desargues’ theorem.

Desargues’ theorem, remember, states that if the lines that join corresponding vertices of two triangles meet in a point, then corresponding sides of the triangle meet in points that lie on a line. The only geometric terms occurring in this theorem are “point”, “line”, “lies on”, “meet in”, and terms that can be defined by means of these terms, such as “being a triangle”. According to the principle of duality, then, for each of these terms there is a dual term: the dual of “point” is “line” and vice versa and the dual of “lies on” is “meet in” and vice versa. Given this translation manual, we can also dualize defined terms, such as “being a triangle” (which is effectively self-dual), and even entire statements, always replacing a term by its dual. Following this recipe, what we end up with in the case of Desargues’ theorem is this:

**Table:** 

Desargues’ theorem	Dual of Desargues’ theorem
If corresponding vertices of two triangles lie on three lines that meet in a point, then corresponding sides of the triangles meet in three points that lie on a line	If corresponding sides of two triangles meet in three points that lie on a line, then corresponding vertices of the triangles lie on three lines that meet in a point

So the dual of Desargues theorem is its *converse* and it is a theorem about the real projective plane just like Desargues theorem itself.

The principle of duality is interesting for a variety of reasons. First, unlike other geometrical theorems it is not directly about points or lines. It is a ‘meta-theorem’ in that it says something about how theorems can be obtained from others by systematically replacing certain terms by others. Second, the principle of duality provides for more economy because for each theorem we get another one for free by simply dualizing. Finally, the principle provides a powerful tool in finding *new* theorems. Indeed, the latter two points were emphasized by nineteenth century geometers as the main virtues of duality.^12^

Perhaps because of the special status of the principle, there was a debate during the first third of the nineteenth century over both its content and proper justification.^13^ Two general approaches can be distinguished that will be particularly relevant for our later discussion. The first can be traced to Poncelet and is based on mappings. The other can be loosely traced to Gergonne, who favoured a more proof-oriented approach.^14^ Let’s start with the latter first. Although Gergonne had no clear view, there is evidence indicating that he thought that the principle of duality was essentially a matter of the symmetric nature of certain basic laws on which projective geometry is ultimately based, which in turn is guaranteed by the presence of elements at infinity. Thus, to prove the dual of a theorem, we may simply use the dualized basic laws. The formal nature of deductive proof then ensures that for any theorem, its dual will be a theorem as well. To emphasize this procedure, Gergonne introduced the convention, later copied by many others, of writing dual theorems as well as their proofs in parallel columns. However, it took some time and more advanced tools, especially explicitly formulated axioms and a clearer conception of proof, before geometers were eventually able to formulate this version of the principle in full clarity. Moritz Pasch seems to have been among the first to do so in his Pasch (1882), where he spends an entire section on the discussion of duality.^15^

Poncelet had a different view on duality. His was based on the theory of poles and polars. The twin concepts of a *pole of a line* and the *polar of a point* had been in use long before Poncelet made the connection to duality.^16^ The idea is this: suppose that we are given some conic section, say, a circle. With each point *P* in the real projective plane, we can then correlate a certain line, called the *polar* of *P*, and with each line *l* a certain point, the *pole* of *l*, relative to the given circle. Specifically, if the point *P* lies outside the circle, construct the tangents on the circle, join the tangency points and you get the polar of *P*. If *P* lies inside the circle, take any two different lines through *P* and construct the tangents of the points where each line intersects the circle. The line determined by the intersection points of corresponding tangents will be the polar of *P*. If *P* lies on the circle, simply take the tangent in *P* as polar. Finally, if *P* is the center of the circle, its polar will be the line at infinity. By reversing these constructions, we can associate with each line a unique point, its pole.

Why is this relevant to duality? By the constructions just described, every point can be correlated with a certain line and every line with a certain point. Moreover, the correlation guarantees that a point *P* lies on a line *l* if and only if the pole of *l* lies on the polar of *P*. More abstractly, the construction thus defines a mapping $\pi$ from points to lines and lines to points such that a point *P* lies on a line *l* just in case $\pi (P)$ passes through $\pi (l)$. Poncelet’s idea, then, was that the possibility of such a correlation guarantees that the principle of duality holds because, via $\pi$, every theorem about points and lines can be transformed into a theorem about the poles and polars corresponding to the lines and points in the theorem.

Both conceptions of duality are important and have precise counterparts in contemporary mathematics. For current purposes, however, we want to focus on the mapping-based conception that goes back to Poncelet and try to understand how it relates to the idea of a reinterpretable language. First, note that if we think of projective geometry as being formulated in a reinterpretable language, then sentences may not only be evaluated with respect to their intended (projective) interpretation, i.e. the real projective plane $\mathbf P$, but also with respect to alternative interpretations of its primitive terms. In particular, we may consider the *dual projective plane* $\mathbf P ^\mathbf{D }$ where the term “point” applies to projective lines, “line” applies to projective points and the relation “lying on” applies to a pair consisting of a line and a point just in case the former passes through the latter. The substance of Poncelet’s argument, then, is that the existence of a function that maps points to lines and lines to points as described earlier guarantees that $\mathbf P$ and $\mathbf P ^\mathbf{D }$ are *isomorphic*. But then, making use of the informal idea that isomorphic structures satisfy the same sentences, it follows that every sentence *S* that is true of $\mathbf P$ must be true of $\mathbf P ^\mathbf{D }$ and vice versa. By definition of the dual projective plane $\mathbf P ^\mathbf{D }$ and the dual $S^D$ of a sentence *S*, this entails that a sentence *S* is true of $\mathbf P$ if and only if $S^{D}$ is true of $\mathbf P$, just as the principle of duality says.^17^

Now, to be sure, we won’t be able to find such a streamlined version of the content and justification of duality in terms of reinterpretable languages, structures, and isomorphisms in the writings of geometers of the nineteenth century. But geometers had been invoking the idea of reinterpretation in connection with duality at times even in the nineteenth century. Arthur Cayley, for example, comments on the principle of duality (or “reciprocity”) in 1854 as follows: “we have only to say that the word ‘point’ shall mean ‘line’, and the word ‘line’ shall mean ‘point’ [$\ldots$] and any theorem [$\ldots$] relating to points and lines will become a corresponding theorem relating to lines and points” (Cayley 1854, p. 246). Cayley’s explicit use of quotation marks and the ‘semantic’ terminology suggests that he thought that the terms “point” and “line” are not tied to their ‘ordinary’ (projective) interpretation, but could be applied to their dual interpretation as well.^18^ In any case, the core of the justification of duality as described earlier seems to have been in place by the turn of the twentieth century. Although Veblen and Young don’t give an explicit proof, given what we’ve said earlier about their conception of axioms as uninterpreted schemas etc., their remark that “the principle of duality may be regarded as a consequence of the presence of correlations” (Veblen and Young 1910, p. 268), is likely intended as an expression of the justification of duality we’ve just described. Another example can be found in a notorious letter written to Frege by David Hilbert, where Hilbert notes that

it is surely obvious that every theory is only a scoffolding or schema of concepts together with their necessary relations to one another, and that the basic elements can be thought of in any way one likes. [$\ldots$] In other words: any theory can always be applied to infinitely many systems of basic elements. One only needs to apply a reversible one-one transformation and lay it down that the axioms shall be correspondingly the same for the transformed things. This circumstance is in fact frequently made use of, e.g.  in the principle of duality, etc., and I have made use of it in my independence proofs. (Frege 1980, pp. 40–41)

On a common reading of the first two sentences of this paragraph, they are a clear expression of the idea that mathematical language is reinterpretable. What is striking about this passage is that Hilbert explicitly draws a connection between this idea of reinterpretation, one-to-one transformations and the principle of duality. Even though we can’t be entirely sure from this passage in isolation, it seems plausible to assume that he had in mind a justification of duality along the lines described earlier.^19^

### Counterexamples and independence arguments

The last topic I want to discuss, perhaps the most important in connection with the emergence of the notion of a reinterpretable language, is the use of counterexamples or ‘models’ in independence arguments. Since much of this is well-known, the following is intended to provide an outline of the bits that are relevant to the discussion of Frege’s views in the next section.

The basic issue here is to establish rigorously that a certain geometrical proposition *cannot* be proved from a group of propositions. Nineteenth century geometry is full of interesting questions of that kind. The most important problem of that sort concerns the *axiom of parallels*.^20^ The axiom of parallels is a planar axiom which says that for each line *l* and each point *P* in the same plane that does not lie on that line, there is a unique line $l'$ through *P* that is parallel to *l*. For centuries geometers had tried to prove the parallel postulate from the remaining ones, without success. By the eighteenth century, several mathematicians therefore started to explore the consequences of negating the parallel postulate, often in an attempt to prove the postulate by *reductio*.^21^ One of them, Johann Heinrich Lambert, had already set out the problem in clear terms in a book posthumously published in 1786. He notes that “the *question itself* concerns firstly *neither the truth nor the conceivability of the Euclidean principle*”. Rather, he continues, “*the question is merely if this principle can be derived correctly from Euclid’s postulates and remaining principles?*” (Lambert 1895, p. 160, 162).^22^ In the first half of the nineteenth century Gauss, Bolyai and Lobachevsky eventually came to accept that the parallel postulate *cannot* be derived from the remaining axioms, that ‘non-Euclidean geometry’ is actually consistent. Yet all of them were lacking a rigorous proof of this. After all, contradiction might still be lurking somewhere further down the line.

Towards the end of the nineteenth century, more sophisticated attempts to provide axiomatic foundations for geometry had led to further independence questions, and new questions in turn made it necessary to be even more careful about the axioms.^23^ Now, we can show that a proposition *S* is provable from propositions $\mathcal {A}$ by simply proving it. But how do we show that it *cannot* be proved from them? Well, what people would do was to devise *counterexamples*, that is, certain constructions in which the propositions in $\mathcal {A}$ were in some informal sense ‘satisfied’ or ‘realized’ or ‘represented’, but *S* was not. For example, to prove that the parallel postulate is independent of the remaining Euclidean principles, various constructions had been devised by Beltrami (1868) and Felix Klein in the Sixties and Seventies of the nineteenth century. Klein referred to such constructions as “realizations” (“Versinnlichungen”) which would be “interpretations of said geometries” (Klein 1871, p. 424, 425). Similar to Lambert before, Klein emphasized that these investigations “do not have the purpose of deciding upon the validity of the axiom of parallels”. Instead, they are “solely meant to address this question: *is the axiom of parallels a mathematical consequence of the other axioms mentioned in Euclid*; to this question these investigations provide a definite *no*.” (Klein 1873, p. 113).^24^

Once again, with hindsight it is easy to understand the use of counterexamples in nineteenth century independence arguments in terms of reinterpretable languages. We think of the proposition *S* to be proved independent and the group of propositions $\mathcal {A}$ which *S* is to be proved independent *of*, as sentences that are formulated in some reinterpretable language. Counterexamples are simply interpretations of such a language for which the statements in $\mathcal {A}$ become true, but *S* becomes false. Then, assuming that provability preserves truth in an interpretation, if *S* were provable from $\mathcal {A}$, then *S* would have to be true in any interpretation in which all the statements in $\mathcal {A}$ are true. Hence, the existence of an interpretation in which all statements in $\mathcal {A}$ are true but *S* is false shows that *S* cannot be provable from the statements in $\mathcal {A}$.

This understanding of the use of counterexamples in independence proofs was well entrenched in the mathematical community at the beginning of the twentieth century. We have already mentioned Hilbert, who used reinterpretations of geometrical terms very consciously and on a large scale in his celebrated (Hilbert 1899) to establish various independence results. Even before Hilbert, others were (almost) equally forthright in using the idea of reinterpretation.^25^ To be sure, people at the time did not yet have a clear conception of logical consequence and related concepts and they were typically relying on an informal notion of proof (like, for example, Hilbert in his (1899)). Despite the growing interest in the foundations of logic and the emergence of the first formal systems of logic by the end of the nineteenth century (like Frege’s Begriffsschrift), the concept of proof as a purely syntactic notion had not yet been adopted by many mathematicians. Even the informal notion of proof was not always clearly separated from ‘semantic’ conceptions of logical consequence and, in contrast to nowadays, people were often using terms like “satisfiability” and “consistency” interchangeably. It took a couple of decades until these notions and their mutual relations had been further clarified by Tarski and others. In any case, what matters for our purposes is that at least some geometers at the turn of the century had thought about the use of counterexamples in independence arguments in terms of reinterpreting parts of mathematical language.

## Frege and reinterpretable languages in nineteenth century geometry

In the previous section we’ve discussed several issues in nineteenth century geometry that gradually came to be understood in terms of reinterpretable languages. In this section I will try to determine how Frege reflected about these matters.

As a reminder, Frege was a trained mathematician and it is apparent from the list of his courses at Jena as well as his published articles where his main fields of interest in non-foundational matters were lying, namely complex analysis and geometry.^26^ So Frege was certainly familiar with the main trends in geometrical research of his time. Indeed, as noted by others, some of Frege’s most celebrated ideas seem to be inspired by his engagement with geometry, particularly projective geometry.^27^ As a trained geometer, then, how did Frege think about the issues discussed in the previous section?

### Frege and elements at infinity

The matter of elements at infinity is discussed by Frege as early as 1873 in his doctoral dissertation titled *On a Geometrical Representation of the Imaginary Forms of the Plane*. Imaginary forms are yet another kind of ‘ideal elements’ that had been introduced into geometry sometime at the turn of the nineteenth century. They naturally arise when we look at geometry from an *analytic* point of view.

To illustrate, take a circle and a straight line. There are three possible positions the circle and the line can have relative to each other. The line can intersect the circle in two points, they can touch each other in one point, or they may not meet at all. Given a suitable coordinate system, we can describe the line and the circle by means of equations, and the problem of determining their relative position comes down to solving a certain quadratic equation that results from substituting the equation of the line into the equation of the circle. If this quadratic equation has two real solutions, the circle and line intersect in two points; if there is one double solution, the line touches the circle at a point; and if there is no real solution, then they are disjoint. Now, the important bit here is that even if there is no *real* solution, there are still two *complex* solutions. So, even in the case where the line and the circle are apparently disjoint, there are still two ‘intersection points’, albeit ones with complex coordinates. In a sense, then, points with real numbers as coordinates form only the visible part of a more inclusive domain.

Of course, imaginary forms are fairly unintuitive creatures. The main aim of Frege’s dissertation is therefore to set up a correlation of imaginary forms of the plane with objects of intuitive, three-dimensional Euclidean space.^28^ Before getting to the main business of the dissertation, Frege makes a few general remarks about ideal elements, comparing imaginary forms with elements at infinity. He writes:

Taken literally, a ‘point at infinity’ is even a contradiction in terms; [$\dots$] The expression is therefore an improper one, and it designates the fact that parallel lines behave projectively like straight lines passing through the same point. ‘Point at infinity’ is therefore only another expression for what is common to all parallels, which is what we commonly call ‘direction’. [$\dots$] By designating the direction as a point at infinity, we forestall a difficulty which would otherwise arise because of the need to distinguish a frequently unsurveyable set of cases according to whether two or more of the straight lines in the set were parallel or not. But once the principle of the equivalence of direction and point is established, all these cases are disposed at one blow. (Frege 1984, p. 1)

Just like most geometers in the nineteenth century, Frege here emphasizes the usefulness of points at infinity for the organization of *Euclidean* geometry (see Sect. 3.1). Frege particularly stresses that the expression “point at infinity” is an “improper” one. It might even seem as if Frege thinks of this kind of talk as a *mere* manner of speaking, having no particular ontological import. But that doesn’t seem to be the case either. A few sentences later Frege notes that points at infinity can be visualized by projecting the plane on a sphere. According to Frege, this would establish a correlation between points of the projective plane and ordinary points on the sphere which has the advantage of bringing the former “before our eyes” (Frege 1984, p. 2). But then again: what is actually visualized by such a “representation”, as Frege calls such correlations? So the question remains: what *are* points at infinity, or, to use Frege’s preferred terminology, what are *directions*?

Frege gives an answer to this question ten years later at a somewhat unexpected place, namely in his *Foundations of Arithmetic* (Frege 1884). Frege’s main concern there is to sketch his logicist project of reducing arithmetic to logic. At a certain point, he contemplates introducing cardinal numbers by means of what is today called an ‘abstraction principle’. The *principle of numerical abstraction* (or ‘Hume’s principle’) may be stated as follows:

***Numerical Abstraction (NA)***. *For all concepts* *F* *and* *G*, *the number of* *F’s is identical with the number of* *G’s iff the* *F’s and* *G’s are equinumerous.*

Here “the number of” is a term-forming operator that attaches to concept terms to form singular terms, and equinumerosity is an equivalence relation between two concepts that is defined in terms of the existence of a bijective mapping between them. Before discussing ***NA***, Frege actually considers various similar principles in paragraphs 64–68, all of them related to geometry. He focuses on one principle in particular, which we might call the *principle of directional abstraction*:

***Directional Abstraction (DA)***. *For all lines* *f* *and* *g*, *the direction of* *f* *is identical with the direction of* *g* *iff* *f* *and* *g* *are parallel.*

Just as in ***NA***, we have again a term-forming operator “the direction of” and an equivalence relation between straight lines, the relation of parallelism. Now, bearing in mind that for Frege points at infinity just *are* directions, what Frege is contemplating here is to introduce points at infinity by way of an abstraction principle. As we noted earlier (and as (Mancosu 2015) has shown in some detail), similar proposals had been made by Theodor Reye and other nineteenth century geometers. However, Frege eventually rejects the idea of introducing abstract objects such as numbers or directions by means of abstraction principles like ***NA*** and ***DA*** because of what came to be known as the ‘Julius-Caesar problem’.^29^ He describes his alternative strategy in §68:

Seeing that we cannot by these methods [abstraction, G.E.] obtain any concept of direction with sharp limits to its application, nor therefore, for the same reasons, any satisfactory concept of Number either, let us try another way. If line *a* is parallel to line *b*, then the extension of the concept “line parallel to line *a*” is identical with the extension of the concept “line parallel to line *b*”; and conversely, if the extensions of the two concepts just named are identical, then *a* is parallel to *b*. Let us try, therefore, the following type of definition:

the direction of line *a* is the extension of the concept “parallel to line *a*”; (Frege 1884, p. 79)

So just as Frege eventually settles for the introduction of cardinal numbers by way of an explicit definition of the number operator in terms of classes (or extensions) of equinumerous concepts, he settles for the introduction of points at infinity by way of an explicit definition of the direction operator in terms of classes (or extensions) of parallel Euclidean lines. Indeed, Frege’s correspondence with Pasch from around 1905 seems to confirm that this was his view throughout most of his career.^30^

What can we learn from this about Frege’s views on reinterpretable languages? The textual evidence seems to suggest that Frege’s views on elements at infinity are plainly at odds with a view on this matter in terms of reinterpretable languages. His view may be summarized as follows: There are *Euclidean* points, *Euclidean* straight lines, and certain relations between these entities which are somehow given to us in intuition. We can then introduce further entities by means of certain logical operations, in particular, by forming *classes*. We may decide to call some of those classes “points at infinity” and group them together with ordinary points. But they are what they are. Frege thus seems to identify the real projective plane with a particular model of the real projective plane. We can visualize or ‘represent’ this particular structure by means of other structures (say, by a sphere) or describe it by means of coordinates. But there is only *one* real projective plane, and so the question of associating different interpretations with words like “point” or “line” does not arise. In fact, Frege seems to deliberately avoid referring to points at infinity *as* ‘points’ and instead prefers to call them ‘directions’. Presumably, this wording is meant to avoid the impression that we have to associate different interpretations with words like “point” in different contexts.

Some of Pasch’s remarks during his correspondence with Frege indicate that Frege indeed rejected the notion that words may refer to different objects in different contexts. In his Pasch (1882), Pasch had formulated basic laws for geometry in which geometrical concepts like “point”, “line”, “between”, etc.  were initially used in their Euclidean meaning. However, later in the book he extends the range of application of these terms to accommodate elements at infinity, noting, for example, that “from now on, the expression ‘proper point’ will mean exactly what was hitherto meant by ‘point’; in this way the unspecified word ‘point’ will be made available for a more general application” (Pasch 1882, p. 40). Similarly for the other primitive notions.^31^ Apparently, Frege had objected to this procedure in an earlier letter to Pasch, which is indicated by Pasch’s remark that “[y]ou will not allow one to talk first about ‘points’ because a ‘point’ is later to be understood as something broader, not just as a Euclidean point” (Frege 1980, p. 106).

Now, we can’t be sure about what Frege’s concerns were exactly. One worry might have been that he thought that Pasch’s procedure was an instance of what Frege criticised under the label *piecemeal definitions*.^32^ By this Frege means the practice of defining a concept on a certain domain of objects (say, the natural numbers) and later redefining it on a broader domain of objects (say, the integers). According to Frege, one of the problems with this procedure is that we can’t be sure that the new definition might not introduce a contradiction. In the case at hand, Frege might have been concerned that some of the definitions introduced for the Euclidean domain may no longer make sense in the wider, projective domain. And, as Frege notes, even if there is in fact no contradiction, “they are not ruled out in principle by this method” (Frege 1903, §57).^33^ A related, but more general, worry that Frege might have had is that in using the term “point” differently in different contexts, Pasch is violating a central requirement on scientific language, the requirement that concepts need to have ‘sharp boundaries’. By this Frege means that a concept has to be definitely either true or false of any given object. Part of the point of this requirement is to rule out ambiguous concepts in scientific language. Hence, since Pasch is using terms like “point” or “line” with different meanings in different contexts, he seems to violate this methodological principle and introduces ambiguity into mathematics. I will come back to this later on. For now, let’s just note that Frege avoids any reference to the notion of a reinterpretable language in the context of the introduction of elements at infinity.

### Frege and the principle of duality

To state the obvious, Frege, as a trained geometer, was aware of the significance of the principle of duality and transfer principles more generally. After the passage from his doctoral dissertation quoted earlier, Frege notes:

By a geometrical representation of imaginary forms in the plane we understand accordingly a kind of correlation in virtue of which every real or imaginary element of the plane has a real, intuitive element corresponding to it. The first advantage to be gained by this is one common to all cases where there is a one-one relation between two domains of elements: that we can arrive at new truths by mere transfer [“Übertragung”] of known propositions. But there is another advantage peculiar to this case: that the non-intuitive relations between imaginary forms are replaced by intuitive ones.^34^

As Jamie Tappenden has observed, it is likely that Frege chose his words with care: the use of the word “Übertragung” is presumably meant to be reminiscent of transfer principles like Hesse’s *Übertragungsprinzip*, a principle that was well-known and recognized to be similar to the principle of duality in relevant respects.^35^ Frege mentions the first advantage, that one-one transformations between two domains of elements give rise to systematic correlations between truths about these domains, almost in passing.

But while Frege apparently appreciates transfer principles and their underlying justification in terms of one-one mappings, there is not much evidence as to how Frege thought about this issue in detail, and there is a only a handful of passages where he mentions duality. There is a passage in his dissertation where he contemplates the idea of generalizing his representation of imaginary forms to cover the projective case as well (Frege 1984, p. 46), and another in a review from 1877, where he briefly discusses the relationship between projective and metrical geometry (Frege 1984, p. 95). There is also a somewhat longer passage in the *Foundations of Arithmetic* where Frege invokes duality in a thought experiment which is supposed to support his view that the meaning of an expression is not to be identified with the subjective ideas associated with that expression (Frege 1884, pp. 35–36). But none of these passages are specific enough to be helpful in understanding how Frege thought about duality more precisely, let alone transfer principles in general.

Interestingly, there is one further episode where Frege was *invited* to elaborate on his views on duality, one-one mappings, and related issues, but where he *didn’t*. The episode is particularly significant in connection with our main issue because it took place in the course of Frege’s famous debate with Hilbert, where the idea of reinterpreting parts of mathematical language was a central issue (more on that shortly). During that debate, Hilbert had written his famous letter to Frege where he states his ‘model-theoretic credo’, noting that

any theory can always be applied to infinitely many systems of basic elements. One only needs to apply a reversible one-one transformation and lay it down that the axioms shall be correspondingly the same for the transformed things. This circumstance is in fact frequently made use of, e.g.  in the principle of duality, etc., and I have made use of it in my independence proofs. (Frege 1980, pp. 40–41)

As mentioned earlier, Hilbert here explicitly connects the principle of duality with one-one transformations and the notion of reinterpretation. Hilbert’s remark can be understood as a *challenge* which may be paraphrased as follows: *Look, Gottlob, what I am doing in my independence proofs—reinterpreting geometrical terms—is something that mathematicians have been doing all along, though perhaps unconsciously. Just look at the the justification of the principle of duality in terms of mappings between dual interpretations, where precisely the same idea is used. So it’s up to you, dear Gottlob, to make sense of practices like these without invoking the notion of reinterpretation.* So what does Frege say in reply to this challenge? He says: “I will reserve the right to reply to what you say about the applicability of a theory and the reversible one-one transformation.” (Frege 1980, p. 48) That’s it.

Earlier, when writing his dissertation, it may have been obvious to Frege that one-one transformations between domains of elements give rise to systematic correlations between truths about these domains. It may not have occurred to him that this might stand in need of further clarification. But here, almost 30 years later, the situation is different. Frege’s reaction to Hilbert comes down to an admission that there was something to be explained. Frege simply doesn’t know how to do that. Even later Frege never made use of his “right to reply” and he never took up the issue in his writings.^36^ So while Frege recognized and appreciated the idea that one-one transformations give rise to transfer principles, he had no idea of how to make sense of this more precisely. And even though Frege did not outright reject Hilbert’s views on the particular issue of duality, Frege certainly had his doubts about invoking the notion of a reinterpretable language in this respect.

### Frege and independence arguments

The last topic discussed in Sect. 3 was the use of counterexamples in independence arguments. Because of Frege’s controversial debate with Hilbert, there is plenty of textual evidence on Frege’s views on this matter in the form of several letters and articles. The following is intended to give an outline of some of the important points of the debate that are related to our main topic.^37^

Immediately after publication of Hilbert’s *Foundations of Geometry* in 1899, Frege had contacted Hilbert, asking for clarification of several points he considered to be problematic. Hilbert had claimed in his *Foundations* that his geometrical axioms would ‘define’ the primitive terms occurring in them. For Frege, this is just plain confusion. The purpose of a definition is to fix the reference of a newly introduced term. Geometrical axioms, on the other hand, are supposed to state certain basic facts about the space of intuition, where it is assumed that the basic terms occurring in them have a reference. Frege’s impression got even worse over the course of the correspondence with Hilbert. In the notorious letter to Frege cited earlier, Hilbert famously claims that “if the arbitrarily given axioms do not contradict one another, then they are true, and the things defined by the axioms exist” (Frege 1980, p. 42). Again, for Frege this is turning the true state of affairs upside down.

One of the central reasons for Frege’s unfavourable impression of Hilbert’s *Foundations* was his feeling that Hilbert had spent too little effort on making precise his underlying methodology, and this impression was reinforced by remarks during their correspondence like the one quoted earlier. Now, Frege acknowledges in the correspondence that in order to prove independence one must “show that the non-satisfaction of one of these axioms does not contradict the satisfaction of the others” by providing counterexamples. “Indeed”, so Frege, “the mutual independence of axioms, if it can be proved at all, can only be proved in this way” (Frege 1980, p. 43).^38^ But while Frege and Hilbert are in agreement about the basic strategy of using counterexamples of some sort to prove independence, this still leaves room for disagreement about how this is to be understood more precisely.

Frege’s final view may be summarized as follows. There are two ways to conceive of axioms, the ‘traditional’ and the ‘Hilbertian’ conception. On the traditional conception, axioms are propositions that express true thoughts about a certain domain which form the basis for all further inferences. Typically these propositions are taken as a basis for a systematization of that domain because they are simpler or more obvious than others.^39^ Hilbertian axioms, on the other hand, are neither propositions nor the thoughts expressed by them. Instead, Frege refers to them as “pseudo-propositions” (“uneigentliche Sätze”) because the primitive terms “point”, “line”, etc.  that occur in them appear to designate specific concepts without actually doing so. Really, on Frege’s view, these words merely “indicate”, that is, they function as variables. Hilbertian pseudo-propositions are therefore expressions of higher-order concepts which become genuine propositions only once the variables are instantiated by meaningful terms (Frege 1984, 309 ff.).

Now, since proving something about Hilbertian axioms does not obviously tell us anything about the real axioms to which they correspond, the question remains how independence is to be proved in the case of axioms in the traditional sense. So has Frege something constructive to say on this question?

Frege provides a sketch of his approach in the last part of the series from 1906. He first emphasizes once again that the objects of independence are *thoughts*, i.e.  what is expressed by sentences. He then goes on to elucidate what he means by “independence” in the realm of thoughts: a thought *G* is independent of a group of thoughts $\Omega$ just in case *G* cannot be reached through a sequence of logical inferences (Frege 1984, p. 339). Frege’s further procedure is curious. He contends that we have to lay down “basic truths” which are supposed to legitimize certain inferences that he deems indispensible if independence is to be proved in a rigorous way. One of them is the basic law that whatever is provable from true premises is itself true (Frege 1984, p. 336). Another is only elucidated informally, but it is critical. Frege calls it an “emanation of the formal nature of logical laws” (Frege 1984, p. 337). Frege describes his approach in Frege (1984, pp. 337–339) as follows: Suppose we want to prove that a certain thought *G*, expressed by a sentence *S*, is independent of a group of thoughts $\Omega$, expressed by a group of sentences $\mathcal {A}$. Frege’s idea is that we “translate” all the relevant sentences into other sentences. He illustrates his proposal by way of two columns of expressions. To each simple expression in the first column there corresponds an expression in the second column. Furthermore, it is assumed that logical expressions are paired with themselves. Sentences in the first column then correspond to sentences in the second column when non-logical expressions are replaced by the corresponding expressions in the second. In this way, a set of sentences $\mathcal {A}$ will be translated into a set $\mathcal {A}^T$, each sentence in $\mathcal {A}^T$ having the same logical form as its corresponding member in $\mathcal {A}$ (remember that logical expressions are not affected by substitution), and *S* will be translated to a sentence $S^T$. Moreover, sequences of sentences will correspond to sequences of sentences. Importantly, since all expressions that are involved are assumed to have both a fixed sense and reference, each sentence will express a specific thought with a particular truth value. Sequences of thoughts expressed by sentences of the first column will therefore correspond to sequences of thoughts expressed by sentences of the other. Frege’s “new law” then amounts to the requirement that *proof sequences* will correspond to *proof sequences*, and independence may be proved as follows:

Let us now consider whether a thought *G* is dependent upon a group of thoughts $\Omega$. We can give a negative answer to this question if, according to our vocabulary, to the thoughts of group $\Omega$ there corresponds a group of true thoughts $\Omega '$, while to the thought *G* there corresponds a false thought $G'$. For if *G* were dependent upon $\Omega$, then, since the thoughts of $\Omega '$ are true, $G'$ would also have to be dependent upon $\Omega '$ and consequently $G'$ would be true. (Frege 1984, p. 339)

Frege expresses some reservations about his own proposal, mainly because of the difficulty of determining “what is proper to logic” and the question of which terms are logical (Frege 1984, p. 339). Indeed, Frege did not take up his proposal again in his later writings.^40^

Frege’s proposal is in many ways idiosyncratic, some might even say confused. As noted by Patricia Blanchette, Frege’s proposal seems to suffer from a peculiar imbalance between his *concept* of independence and the proposed method of *proving* independence. While Frege’s concept of independence is conceived as a relation between *thoughts*, which on Frege’s view are language-independent entities, his method for *proving* independence is based on the syntactic operation of substitution of *expressions*. As Blanchette has argued, this seems to have the effect that, in general, Frege’s method is not fit to serve its intended purpose.^41^ Still, Frege’s discussion of Hilbert’s independence proofs as well as his own proposal contain several interesting ideas. I have to confine myself to two points that are relevant to our main topic.^42^

First, Frege’s proposal provides an understanding of the use of counterexamples which, as far as I can see, is fully compatible with most mathematician’s actual practices regarding independence arguments in the late nineteenth century. In particular, on Frege’s account (perhaps against his own intentions) the question of independence becomes a matter of purely formal features of the sentences involved, just as on the Hilbertian conception. But in spite of this, Frege’s proposal is still an alternative. Rather than *reinterpretation*, the effective notion in Frege’s approach is that of *substitution* of fully interpreted terms. Moreover, the two accounts may even differ extensionally, depending on how the use of substitutions is understood more precisely.^43^ In any case, it seems appropriate to me to treat Frege’s approach as a genuine, informal alternative to Hilbert’s. It certainly is with respect to the use of the notion of a reinterpretable language.

The second point I want to mention is related to an issue we’ve touched on earlier. As Jamie Tappenden has first pointed out (Tappenden 2005), Frege’s presentation of his method in terms of two columns, where each term of one column corresponds to a term in the other, sentences correspond to sentences, and so on, bears a striking resemblance to standard presentations of the principle of duality in nineteenth century projective geometry. As we saw (see Sect. 3.2), on *one* understanding of the principle of duality, loosely dating back to Gergonne, it is understood as a principle about dual theorems whose justification is based on the fact that each basic truth of projective geometry has a dual, and that logical deduction is purely formal. So even though Frege nowhere mentions duality explicitly in his articles on the foundations of geometry, Tappenden may well be right when he notes that “Frege could not have failed to be aware that projective duality was an evident realization of the ‘new basic law’ he was describing” (Tappenden 2005, p. 214). In fact, Tappenden suggests in Tappenden (2000, p. 273) that Frege’s 1906 proposal contains something like an oblique (and somewhat delayed) response to the challenge posed by Hilbert in his letter to Frege from six years earlier (see Sect. 4.2).^44^ Note though that, on their most natural reading, Hilbert’s remarks are concerned with the *second* understanding of the principle of duality we have discussed, the one that dates back to Poncelet, where duality is explained in terms of one-one mappings between points and lines. So even if we take it for granted that Frege’s 1906 proposal contains an implicit account of duality, the issue raised by Hilbert concerning the mapping-based conception of duality is not addressed. Ironically, then, if Tappenden is right with his observation that there is a close connection between Frege’s proposal concerning independence proofs and the principle of duality, then this actually provides evidence *against* (parts of) his general narrative, because then the only way for Frege to make sense of duality is in terms of *formal proofs*, not in terms of (however informal) ‘model theoretic’ reasoning.

There is much more to be said on Frege’s proposal concerning independence proofs, which lack of space prevents me from doing. What the discussion should have established is that in the case of independence proofs too, Frege clearly avoids the idea of reinterpretation. As he says quite explicitly in the 1906 series to Alwin Korselt, Hilbert’s proxy: “The word ‘interpretation’ is objectionable, for when properly expressed, a thought leaves no room for different interpretations.” (Frege 1984, p. 315)

## Discussion

We have seen that for a variety of issues in nineteenth century geometry that *can* be conceptualized in terms of reinterpretable languages, and that *have been* conceptualized in such a way by some by the turn of the twentieth century, Frege did not do so. In fact, at several points he explicitly rejects the idea of associating different interpretations with a term. Why is that?

It seems to me that the main ingredients of an answer to this question are fairly transparent (although they raise further questions). First of all, Frege apparently did not feel any *need* to invoke the notion of a reinterpretable language in any of the issues we’ve discussed. Although he did not have fully worked out accounts, he thought that issues like the ones discussed in Sect. 3*can* be dealt with within the confines of a ‘traditional’, fixed-interpretation conception of mathematical language. No appeal to a reinterpretable language *has to* be made, and therefore no appeal *should* be made.^45^

A second minor reason for Frege’s resistance to the notion of reinterpretation, which comes out most pointedly during his debate with Hilbert, may be traced to the choice of words of his opponents. Frege certainly felt offended by Hilbert’s sweeping remarks about mathematical theories being “schemas” that “can be applied to infinitely many systems of things”. Hilbert’s defender Korselt also tends to speak of Hilbert-style axiom systems as “formal theories” or “purely formal systems” (Kluge 1971, p. 40). Presumably, this way of talking triggered Frege’s notorious aversion to formalist conceptions of mathematics.^46^ Ultimately, I don’t think that Frege took Hilbert to be a follower of the crude kind of formalism that Frege was opposing so vigorously throughout his career (mathematical statements as meaningless marks, mathematics as a mere game, etc.). Still, although most of Frege’s objections to Hilbert are not directed against formalism as such, Frege might have sensed some sort of spiritual connection between the notion of a reinterpretable language and formalist philosophies.

I think the ultimate reason why Frege rejected the notion of a reinterpretable language is fairly mundane. He rejects it because the only way he can make sense of it is in terms of *ambiguity*, which is a defect that has to be avoided. From Frege’s perspective, to *reinterpret* a mathematical term is to deliberately introduce ambiguity into mathematics. Given that Frege spent much of his career on developing an artificial language intended precisely to avoid ambiguity, it shouldn’t come as a surprise, then, that he rejects this notion. Frege is quite frank about the connection he sees between the method of reinterpretation and ambiguity on a number of occasions throughout his articles on the foundations of geometry. To give just a few examples, in Frege (1984, p. 306) he notes that “the appearance that ambiguous signs are necessary arises from unclear thinking and insufficient logical insight”. The same point is made when he writes that “[t]he word ‘interpretation’ is objectionable, for when properly expressed, a thought leaves no room for different interpretations. We have seen that ambiguity simply has to be rejected and how it may appear to be necessary because of insufficient logical insight” (Frege 1984, pp. 315–316). In the same piece he writes that “there must be no ambiguity” (Frege 1984, p. 311), and that “the univocity of signs—which we must retain at all cost-excludes different interpretations” (Frege 1984, p. 318). The list could be continued. As we saw in Sect. 4.1, worries about ambiguity may have also been among the reasons for rejecting the notion of a reinterpretable language in connection with elements at infinity.

It is worth stressing that nothing that Frege says indicates that he thinks that it is somehow ‘impossible’ to think of an expression as having another sense (or reference, interpretation) than the one it actually has. After all, which sense (or reference, interpretation) is attached to an expression is a complicated human affair and to a large degree a matter of conventions. Rather, Frege’s point is a *normative* one: we *should* not deliberately attach different interpretations to a word, certainly not in a language that is supposed to be adequate for scientific purposes. So even if the notion of a reinterpretable language would somehow bear an advantage of allowing a kind of ‘useful ambiguity’ that may be beneficial for certain purposes, it would still remain a defect.^47^

Let me in conclusion make a few tentative remarks concerning the controversy over Frege’s stance towards ‘metatheoretic’ questions. As mentioned in the introduction, there is a real danger of anachronism in speculating about Frege’s views on ‘metatheoretical’ questions, and ‘semantics’ in particular. ‘Metatheory’ as we understand it today is the result of various developments that Frege could not have foreseen, like, for instance, the development of set theory as an independent discipline, the emergence of first-order logic as a distinguished system, or the discovery of limitative results such as Tarski’s or Gödel’s.^48^ Still, the discussion in the previous sections should have confirmed what people like Patricia Blanchette, Aldo Antonelli, Robert May, Jamie Tappenden, and others have emphasized before: that Frege was very much interested in a variety of questions that ought to be considered as ‘metatheoretic’ on any reasonable, non-anachronistic, understanding of this term. He was certainly familiar with, and making use of, transfer principles like duality; he was interested in methodological questions concerning the foundations of projective geometry, including the proper treatment of elements at infinity; and he made a serious attempt to explicate the use of counterexamples in independence proofs.

However, the discussion should have also established (or confirmed) that Frege had serious qualms with forms of reasoning that involve the notion of a reinterpretable language. So if this notion is taken to be central to informal model-theoretic reasoning, then people like James Conant, Tom Ricketts, or Jaakko Hintikka are right. But while I think that this is a significant point, it also requires qualification. First, semantic reasoning is not necessarily model-theoretic reasoning. Even if Frege rejected ‘model-theoretic reasoning’, this does not rule out semantics altogether, understood as the scientific study of concepts like truth, reference, etc. Second, while Frege might have rejected informal model-theoretic reasoning in the narrow sense that involves reinterpretation, this does not entail that Frege had problems with model-theoretic reasoning on *any* plausible, non-anachronistic conception of that term. Indeed, there are more liberal conceptions of this notion that are arguably more useful if we want to understand the actual history of model theory. Much of the work on formal axiomatics within a type-theoretical framework that was done during the Twenties and Thirties of the twentieth century would not qualify as being concerned with informal model theory if the idea of reinterpretation was strictly required for this, despite the fact that this work was certainly significant for the actual development of model theory. A broader conception of model-theoretic reasoning that includes such developments will presumably have to include some of Frege’s ideas as being concerned with ‘informal model-theoretic reasoning’ as well. As indicated in Sect. 4.3, Frege *did*, for example, provide a reconstruction of Hilbert’s methodology relating to independence proofs (primitive terms as variables, axiom systems as higher-order concepts, etc.) that comes quite close to the type-theoretical reconstructions proposed later, e.g.  by Carnap.^49^ Furthermore, as we saw in Sect. 4.2, Frege was familiar with the notion that one-to-one mappings between domains of elements induce systematic correlations between truths about these domains. And while Frege had no clear conception of how this works in detail and certainly did not invoke the notion of reinterpretation in this respect, it is also not hard to make out connections to later developments in model theory.^50^ Now, Frege may not have played a prominent role in the development of model theory. But I think that it’s fair to say that, at the very least, he wasn’t totally cut off from the developments that eventually led to the emergence of model theory either. However, fleshing this out is work for another paper.

## Footnotes

1 A note on quotation marks: I use single quotation marks within quotations and as scare quotes and double quotation marks to refer to expressions and for proper quotations.

2 Standard expositions of modern model theory can be found in Chang and Keisler (1973) and Hodges (1993). The emergence of the notion of a *formula being true in a structure*, especially in Tarski’s work, is dicussed in Hodges (1986). The conceptual structure and prehistory of model theory are discussed in Demopoulos (1994) and Hintikka (1988), with a critical eye on Frege.

3 For more on the great transformations in mathematics at the turn of the twentieth century in general, see Gray (2008) and the third volume of Kline (1972). Developments in geometry in particular are discussed in the classic Freudenthal (1962) and Gray (2007).

4 The origins of projective geometry are discussed in Kline (1972, ch. 14). A detailed study of the prehistory of projective geometry can be found in Andersen (2007). Andersen also critically discusses the idea that points at infinity originated in the study of perspective in Andersen (2007, pp. 402–403) and Andersen (2007, p. 441).

5 Since it is possible to prove the converse of Desargues’ theorem from Desargues’ theorem itself (and conversely), the biconditional consisting of Desargues’ theorem and its converse is sometimes referred to as “Desargues’ Theorem”. Theorem 1$^*$ is sometimes referred to as the *affine version* of Desargues’ theorem. For various proofs as well as philosophical and historical discussion, see Arana and Mancosu (2012) and Dawson (2015, pp. 93–110).

6 Theodor Reye, for example, writes in his *Die Geometrie der Lage* that “this view has the advantage over the old view that many propositions can now be stated in full generality, whereas otherwise exceptions would have to be mentioned, and many seemingly different propositions can now be covered by a single proposition” (Reye 1866, p. 15, transl. by author), similarly Von Staudt (1847, p. 25). Arthur Cayley, having discovered that a metric can be defined in a purely projective setting if we fix some conic as ‘absolute’, notes that “[m]etrical geometry is thus a part of descriptive [i.e.  projective, G.E.] geometry, and descriptive geometry is *all* geometry and reciprocally” (Cayley 1859, p. 90). See Gray (2007, ch. 22) for further discussion.

7 Points at infinity had often been referred to as ‘infinitely distant points’. This triggers the idea that somehow these points have an ‘infinite distance’ from each ordinary point, which certainly *sounds* odd. See e.g.  Reye (1866, p. 15) or the discussion in Von Staudt (1847, 23 ff.).

8 An excellent discussion of ‘abstractionist’ proposals in nineteenth century geometry in connection with elements at infinity can be found in Mancosu (2015), where Mancosu discusses the mathematical context of Frege’s celebrated definition of numbers in his Frege (1884). We will return to this issue in Sect. 4.

9 See Gray (2007, chs. 13–16) for a discussion of this algebraic approach to projective geometry.

10 Pasch notes with respect to the transition from Euclidean to projective geometry that “from now on the expression ‘proper point’ will mean exactly what was hitherto meant by ‘point’; in this way the unspecified word ‘point’ will be made available for a more general application” (Pasch 1882, p. 40). See Schlimm (2010) for more on Pasch’s philosophy of mathematics.

11 There is a corresponding principle for solid projective geometry. Here, for every theorem there is another theorem which results from the first by replacing the term “point” with “plane” and vice versa. The term “line” is its own dual. The importance of duality for the development of modern logic is especially emphasized in Nagel (1939). See also Tappenden (2005, pp. 190–194) for further discussion of duality and transfer principles more generally.

12 In a discussion of the principle of duality, Otto Hesse notes in (1866, p. 32, transl. by author) that for these reasons “transfer principles are of much greater importance in geometry than single theorems”. For further discussion see again (Tappenden 2005, p. 193).

13 For a discussion of the main mathematical points see Gray (2007, pp. 53–62). The polemical nature of the controversy is discussed in Lorenat (2015).

14 See Poncelet (1822), Gergonne (1825), and Nagel (1939, 179 ff.) for discussion. Specker (1958) discusses both versions from a contemporary viewpoint which is informed by modern formal logic. A third ‘algebraic’ approach to duality in terms of coordinates had been developed by Julius Plücker in his Plücker (1831).

15 Pasch discusses this proof-oriented conception and justification of duality in section 12 of his Pasch (1882), where he also stresses the purely formal nature of deductive proof in geometry, noting that “if geometry is to be genuinely deductive, the process of inferring must everywhere be independent of the sense of the geometrical concepts, just as it has to be independent of the figures” (Pasch 1882, p. 98). However, he also notes that the justification in terms of basic laws (“Grundsätze”) is of limited scope (Pasch 1882, p. 94) and that the principle of duality is established by this procedure for the geometry of position only “insofar as it can be developed from [the] basic laws [in §§7, 8, 9]” (Pasch 1882, p. 95). Thirty years later, Veblen and Young, referring to this proof-oriented conception, note that the “method of formal inference from explicitly stated assumptions” makes duality “almost self-evident” (Veblen and Young 1910, p. 29). See also Eder and Schiemer (2018) for a discussion of duality in nineteenth century geometry.

16 See Gray (2007, ch. 2, 5) and Kline (1972, ch. 35) for further discussion of the theory of poles and polars.

17 In the context of projective geometry, an isomorphism between a projective plane and its dual is also called a *correlation*. A correlation $\pi$ with the additional property that $\pi (\pi (P)) = P$ and $\pi (\pi (l)) = l$ (such as the function $\pi$ mentioned above) is called a *polarity*. The argument just sketched can be made precise by making use of the isomorphism lemma, which is a basic result in model theory that states that isomorphic structures are elementary equivalent, i.e.  satisfy the same sentences.

18 Cayley did not, however, provide a rigorous proof of duality there. Given his strong background in the analytic tradition of projective geometry, it seems likely that he thought of duality in terms of coordinates in a way that is similar to the one proposed by Plücker (1831, pp. v–ix).

19 Unfortunately, in the edition of Hilbert’s lectures on geometry (Hilbert 2004), the passages where he discusses duality have been left out. Ralf Haubrich reports in his introduction to the lectures on projective geometry from 1891 that Hilbert adopted his proof of duality from Reye’s *Geometrie der Lage*, which is based on the theory of poles and polars. See his discussion in Hallett (2004, pp. 19–20).

20 See Gray (2007, ch. 7–10) and Kline (1972, ch. 36) for more on the historical and mathematical background relating to the axiom of parallels and the emergence of Non-Euclidean geometries.

21 There are two ways to negate the parallel postulate. Given some line, there may be *no* parallels through a point not on that line, or *more than one*. The former leads to ‘elliptic geometry’, the latter to ‘hyperbolic geometry’.

22 Lambert is quite explicit in that in dealing with this question “we may wholly ignore $\ldots$ the representation of the subject matter. Since Euclid’s postulates and remaining axioms are stated in words, we can and should demand that no appeal be made anywhere in the proof of the matter itself, but that the proof be carried out $\ldots$ in a thoroughly symbolic fashion” (Lambert 1895, p. 162). See Webb (1995, p. 3) for discussion of this passage (with minor differences in the translation).

23 For example, during the 1890s, Hilbert had become interested in Desargues’ theorem, making the observation that even though it is a planar incidence theorem, all of its standard *proofs* were relying on either spatial or metrical notions. Hilbert’s observation naturally lead him to ask whether Desargues’ theorem could be proved using only basic *planar* truths. As it turned out, the answer is ‘no’. Independence arguments related to Desargues’ theorem had been put forward even before Hilbert in work by Peano. See Arana and Mancosu (2012), Dawson (2015, 107 ff.), and Hallett (2008) for further discussion. Further independence problems were studied in an axiomatic setting by various Italian geometers in the 1890s which also led to the consideration of finite geometries. A discussion of the Italians and their contributions to the foundations of (projective) geometry can be found in chapters 24 and 27 of Gray (2007).

24 For a historical discussion of the use of ‘models’ in geometry and their role in the development of modern formal logic see also Blanchette (2017), Nagel (1939), and Webb (1995). Arana and Mancosu (2012, §3) provide a detailed discussion of independence arguments relating to Desargues’ theorem.

25 Once again, the Italians seem to have been the first. See, for example, Fano 1892 and Peano (1894) and Freudenthal (1962) for discussion. It should also be mentioned that, in spite of passages like the one quoted earlier, there is still room for disagreement about how Hilbert understood his method of proving independence and consistency in detail at various times. See Eder and Schiemer (2018) for further discussion.

26 Frege held introductory courses in complex analysis and seminars on advanced topics such as elliptic functions and Abelian integrals, and in geometry he lectured on both synthetic and analytic geometry. See Kreiser (2001, p. 280). His non-foundational articles too were mainly concerned with geometry. Among the published writings on geometry, we find his dissertation *On a Geometrical Representation of Imaginary Forms in the Plane* (1873), *Lecture on a Way of Conceiving the Shape of a Triangle as a Complex Quantity* (1878), *Lecture on the Geometry of Points in the Plane* (1884), as well as several reviews. All these works are reprinted in Frege (1984).

27 See Mancosu (2015), Tappenden (2005, 2006), and Wilson (1992, 2010) for more on Frege’s mathematical setting and its influence on his foundational work on logic and arithmetic.

28 The problem here is that if complex numbers are identified with pairs of real numbers (the real and imaginary parts), then the two-dimensional complex plane will be four-dimensional. To overcome this problem, Frege introduces two distinguished planes in three-dimensional Euclidean space, the *plane of the real* and the *plane of the imaginary*, which are assumed to be parallel, say, the *xy*-plane and some parallel plane above the *xy*-plane. Given some imaginary point *P* with coordinates $\xi + i \xi '$ and $\eta + i \eta '$, we can then identify the pair $(\xi , \eta )$ consisting of the real parts and the pair $(\xi ', \eta ')$ consisting of the imaginary parts with a point on the plane of the real and a point on the plane of the imaginary respectively. Since these two points will determine a straight line, the imaginary point *P* can be ‘represented’ in three-dimensional space by this straight line. After describing the basic setup, Frege goes on to investigate what imaginary curves (including straight lines) will look like according to this basic construction (and given the way in which these curves are defined algebraically). See Shipley (2015) for further discussion of Frege’s dissertation.

29 Roughly, the problem is that principles like ***NA*** and ***DA*** do not settle the truth values of all identity statements. Specifically, if $\mathsf {Num}$ represents the number-of-operator, then identity statements of the form “$\mathsf {Num}(F) = c$” are not settled unless *c* itself is of the form $\mathsf {Num}(G)$ for some concept *G*. An analogous problem obviously arises for directions. See Heck (1997) for a more detailed discussion of the Caesar problem.

30 Unfortunately, most of Frege’s letters to Pasch are lost. According to the editors of Frege’s correspondence, a fragmentary draft of a letter to Pasch survived in which Frege essentially describes the position sketched here. See footnote 1 in Frege (1980, p. 106). However, we can also reconstruct Frege’s position from Pasch’s replies. See Frege (1980, p. 106).

31 The situation in Pasch (1882) is actually more complex since Pasch usually reformulates statements that involve points at infinity in terms of “bundles of lines”. Still, the general point stands.

32 See Frege (1903, §§56–58), English translation in Frege (2016) and Blanchette (2012, 62 ff.) for a discussion of piecemeal definition. I am grateful to Jeremy Heis for calling my attention to the issue of piecemeal definitions as a possible source for Frege’s troubles in the Pasch correspondence.

33 This reading seems to be indicated also by Pasch’s remark that “I think your intention, which I find perfectly evident, will also be fulfilled if one talks first about ‘points’ without qualification and then extends the application of the word ‘point’ while stating that in all preceding propositions and definitions the word ‘point’ is to be understood only in the original sense”.

34 See Frege (1984, pp. 2–3). I am following Jamie Tappenden’s translation of “bloße Übertragung” by “mere transfer of” instead of “merely carrying over”, and for the same reasons. See footnote 5 in his Tappenden (2005, p. 222).

35 Hesse had introduced the principle in an article titled “Ein Übertragungsprincip” (“A transfer principle”). See Hesse (1897, 531–538). The principle states a correlation between truths about the complex projective plane and truths about point pairs on the complex projective line. Hesse explicitly compares his *Übertragungsprinzip* to the principle of duality (Hesse 1866, 31 ff.). For further discussion, see Tappenden (2000, 2005, pp. 190–194).

36 On this point I disagree with Jamie Tappenden, who seems to think that Frege indeed took up the issue in his article series on geometry in 1906. See Tappenden (2000, p. 273). I will come back to this in Sect. 4.3.

37 Since Hilbert refused to agree to publish their correspondence, Frege put forth his views in an article series in 1903 and another series in 1906. The 1906 series is partly addressed to Alwin Korselt, who had been defending Hilbert’s approach. The articles can be found in Frege (1984). They are also reprinted in Kluge (1971), together with Korselt’s article and the relevant correspondence between Frege and Hilbert. The *locus classicus* in the secondary literature concerning the Frege–Hilbert controversy is Resnik (1974). More recent studies are e.g.  Blanchette (1996), Blanchette (2012, 108 ff.), Demopoulos (1994), Hallett (2010), and Wehmeier (1997).

38 As Frege is quick to point out, though, he is adopting to Hilbert’s terminology concerning ‘axioms’ in these passages. This is important because in his later published writings Frege strictly distinguishes between the *Hilbertian* conception of axioms as conditions or higher-order concepts and axioms in the ‘traditional’ sense.

39 Frege is somewhat ambiguous in his use of the term “proposition” (“Satz”) in his articles on the foundations of geometry. He says, for example, that “[w]hat I call a proposition *tout court* or a real proposition [“Satz schlechtweg oder eigentlichen Satz”] is a group of signs that expresses a thought; however, whatever has only the grammatical form of a proposition I call a pseudo-proposition [“uneigentlichen Satz”]” (Frege 1984, p. 308), indicating that a real proposition is something like a sentence-thought pair. In another passage of the same article series, he says “When one uses the phrase ‘prove a proposition’ in mathematics, then by the word ‘proposition’ one clearly means not a sequence of words or a group of signs, but a thought” (Frege 1984, p. 332), indicating that the thought itself is indeed *all* that’s relevant. Frege comes back to this issue in an unpublished manuscript from 1914 (Frege 1979, p. 206).

40 In his notes on an article by Jourdain, Frege even claims that the “indemonstrability of the axiom of parallels cannot be proved. If we do this apparently, we use the word ‘axiom’ in a sense quite different from that which is handed down to us” (Frege 1980, p. 183). Some scholars think that this shows that Frege’s 1906 sketch of independence proofs was merely a slip of the pen. See, for example, Ricketts (1997). An alternative view is defended by Jamie Tappenden (2000).

41 Since formal derivability is purely a matter of the formal properties of sentences, Frege’s substitution-based method guarantees that a sentence *S* cannot be derived (in some sound formal calculus) from a group of sentences $\mathcal {A}$ whenever there is a suitable sequence of terms that translates the sentences in $\mathcal {A}$ into true sentences $\mathcal {A}^T$ and *S* into a false sentence $S^T$. However, this does *not* guarantee that the *thought* expressed by *S* cannot be proved from the thoughts expressed by the sentences in $\mathcal {A}$. In general, a thought can be expressed in a number of different ways, and different sentences may reveal different derivability-relations. See Blanchette (1996, 2007), and chapter 5 in Blanchette (2012) for a detailed discussion.

42 See Antonelli and May (2000) and Ricketts (1997) for more on Frege’s ‘new science’ and Eder (2016) for a discussion with a particular focus on the ‘axiomatic’ aspect of Frege’s approach to independence proofs.

43 If substitution sequences are assumed to be drawn from some *fixed* language, then, no matter how rich the language, there will always be interpretations that do not correspond to sequences of expressions from this language. (This is due to the elementary fact that there are more interpretations than expressions of some fixed language.) Thus, it might happen that there is a set of sentences $\mathcal {A}$ and a sentence *S*, such that *S* is declared independent of $\mathcal {A}$ by Hilbert’s account, but not by Frege’s, because there are no expressions available that correspond to the interpretation that witnesses the Hilbertian independence of *S* from $\mathcal {A}$. In connection with Frege’s proposal concerning independence proofs, this has been mentioned already by Michael Resnik in his (1974). More recently, Patricia Blanchette notes in Blanchette (2012, p. 126) that “as long as the language in question contains names for all of the objects and relations (or their extensions) to which Hilbert has recourse in constructing interpretations, Hilbert’s independence-test and [Frege’s] proposed 1906 independence-test will have exactly the same results”. Note that the equivalence between Hilbert’s and Frege’s method follows if the underlying assumption is that *for every Hilbertian interpretation we can find a language in which this interpretation can be expressed*, just as Blanchette claims. However, the equivalence does *not* follow if the underlying assumption is that *there is a fixed language in which every Hilbertian interpretation can be expressed*, because such a language plainly does not exist (if we assume that Hilbert has recourse to *every* set-theoretical interpretation). Now, I am not suggesting that Frege’s account necessarily relies on a fixed language from which substitution sequences are drawn. But I can’t see anything to the contrary in Frege’s suggestions either.

44 Tappenden writes that “[i]n response to Hilbert’s direct question [about one-one transformations and duality], Frege indicates that he hasn’t addressed this issue but he reserves the right to do so” and he notes in brackets that “Frege indicates no answer before 1906”, suggesting that Frege *did* indicate an answer in 1906.

45 Similar remarks apply to other developments in nineteenth century mathematics that seem to require a point of view that allows for reinterpretability, like the emergence of abstract algebra. In a letter to Edward V.  Huntington from around 1902, Frege talks about his take on ‘algebraic’ axiom systems (Frege 1980, pp. 58–59) and proposes a reconstruction of such systems similar to the higher-order reconstruction that was proposed in connection with Hilbert.

46 This connection is also made e.g.  by Eike-Henner Kluge in the introductory remarks of the compilation (Kluge 1971), in which Frege’s articles on the foundations of geometry are published together with his articles on formalist conceptions of arithmetic.

47 Antonell and May (2000) have claimed that Frege’s ‘fixed interpretation conception of language’ would be a consequence of his doctrine of sense and reference. Since a proper language is understood by Frege as a system of signs, and a sign consists of an expression together with a sense, it would follow from Frege’s doctrine that sense determines reference that an expression of a proper language is ‘immutably equipped’ with a fixed reference or interpretation. Thus, changing the sense (and hence the reference) attached to an expression, would amount to changing the entire language. While this seems to be a fair description of Frege’s views, it doesn’t explain why reinterpretation, understood as a systematic ‘change of language’ that may be useful for certain purposes, should be ruled out.

48 See Awodey and Reck (2002) and Badesa et al. (2009) for a discussion of the development of logic at the beginning of the twentieth century.

49 Cf.  Carnap (2000). For more on Frege’s reconstruction, see the classic (Resnik 1974). For further discussion of type-theoretical reconstructions of ‘model theory’, see Schiemer and Reck (2013) and Schiemer et al. (2017). Even issues like the *categoricity* of a system of axioms (or ‘higher-order concepts’) can be understood within such a framework. Indeed, Frege seems to have been alert of these kind of issues, which is indicated, for example, by his theorem 263 in the *Grundgesetze*, where he essentially establishes the categoricity of his system of ‘axioms’ for arithmetic. For more on Frege’s ‘categoricity proof’, see Heck (1993).

50 See again (Tappenden 2005) for further discussion.

## References

[CR1] Andersen K (2007). The geometry of an art: The history of the mathematical theory of perspective from Alberti to Monge.

[CR2] Antonelli A, May R (2000). Frege’s new science. Notre Dame Journal of Formal Logic.

[CR3] Arana A, Mancosu P (2012). On the relationship between plane and solid geometry. The Review of Symbolic Logic.

[CR4] Awodey S, Reck E (2002). Completeness and categoricity, part 1: Nineteenth-century axiomatics to twentieth-century metalogic. History and Philosophy of Logic.

[CR5] Badesa C, Mancosu P, Zach R, Haaparanta L (2009). The Development of mathematical logic from Russell to Tarski, 1900–1935. The development of modern logic.

[CR6] Beltrami E (1868). Saggio di interpretazione della geometria non-euclidea. Giornale di Mathematiche.

[CR7] Blanchette P (1996). Frege and Hilbert on consistency. Journal of Philosophy.

[CR8] Blanchette P (2007). Frege on consistency and conceptual analysis. Philosophia Mathematica.

[CR9] Blanchette P (2012). Frege’s conception of logic.

[CR10] Blanchette, P. (2017). Models in geometry and logic: 1870–1920. In S. S. Niniiluoto (Ed.), *Logic, methodology and philosophy of science: Proceedings of the 15th international congress* (pp. 41–61). College Publications.

[CR11] Carnap R (2000). Untersuchungen zur Allgemeinen Axiomatik.

[CR12] Cayley A (1854). An introductory memoir upon quantics. Philosophical Transactions of the Royal Society of London.

[CR13] Cayley A (1859). A sixth memor upon quantics. Philosophical Transactions of the Royal Society of London.

[CR14] Chang CC, Keisler HJ (1973). Model theory.

[CR15] Conant J (1992). The search for logically Alien thought: Descartes, Kant, Frege and the tractatus. Philosophical Topics.

[CR16] Dawson JW (2015). Why prove it again? Alternative proofs in mathematical practice.

[CR17] Demopoulos W (1994). Frege, Hilbert, and the conceptual structure of model theory. History and Philosophy of Logic.

[CR18] Eder G (2016). Frege’s ‘On the foundations of geometry’ and axiomatic metatheory. Mind.

[CR19] Eder G, Schiemer G (2018). Hilbert, duality, and the geometrical roots of model theory. Review of Symbolic Logic.

[CR20] Fano G (1892). Sui postulati fondamentali della geometria proiettiva. Giornale di Mathematiche.

[CR21] Frege G (1884). The foundations of arithmetic.

[CR22] Frege, G. (1903). *Grundgesetze der Arithmetik* (Vol. II). Jena: Verlag von Hermann Pohle. edited and translated into English in Frege 2016.

[CR23] Frege G (1979). Posthumous writings.

[CR24] Frege G (1980). Philosophical and mathematical correspondence.

[CR25] Frege G (1984). Collected papers on mathematics, logic and philosophy.

[CR26] Frege, G. (2016). *Basic laws of arithmetic* (vol. I & II). Oxford: Oxford University Press. edited and translated by Philip A. Ebert and Marcus Rossberg, with Crispin Wright, 2013.

[CR27] Freudenthal H, Nagel E, Suppes P, Tarski A (1962). The main trends in the foundations of geometry in the 19th century. Logic, methodology, and philosophy of science.

[CR28] Gergonne, J. D. (1825/26). Considérations philosophiques sur les éléments de la science de l’éntendue. *Annales Math. Pur. Appl. **16*, 209–231.

[CR29] Gray J (2007). Worlds out of nothing: A course in the history of geometry in the 19th century.

[CR30] Gray J (2008). Plato’s ghost: The modernist transformation of mathematics.

[CR31] Hallett M, Mancosu P (2008). Reflections on the purity of method in Hilbert’s Grundlagen der geometrie. The philosophy of mathematical practice.

[CR32] Hallett M, Potter M, Ricketts T (2010). Frege and Hilbert. The Cambridge companion to Frege.

[CR33] Heck RK (1993). The development of arithmetic in Frege’s Grundgesetze der Arithmetik. The Journal of Symbolic Logic.

[CR34] Heck RK, Heck RK (1997). The Julius Caesar Objection. Language, thought, and logic: Essays in honour of Michael Dummett.

[CR35] Hesse O (1866). Vier Vorlesungen aus der Analytischen Geometrie.

[CR36] Hesse O (1897). Gesammelte Werke.

[CR37] Hilbert, D. (1899). *Grundlagen der Geometrie*. Leipzig: Teubner, 1968, 10th edition.

[CR38] Hilbert D (2004). David Hilbert’s lectures on the foundations of geometry 1891–1902.

[CR39] Hintikka J (1988). On the development of the model-theoretic viewpoint in logical theory. Synthese.

[CR40] Hodges W (1986). VIII: Truth in a Structure. Proceedings of the Aristotelian Society.

[CR41] Hodges W (1993). Model theory.

[CR42] Klein F (1871). Über sogenannte Nicht-Euklidische Geometrie. Nachrichten von der Königl. Gesellschaft der Wissenschaften und der Georg-Augusts-Universität zu Göttingen.

[CR43] Klein F (1873). Über sogenannte Nicht-Euklidische Geometrie (2). Mathematische Annalen.

[CR44] Kline M (1972). Mathematical thought from ancient to modern times.

[CR45] Kluge E-HW (1971). On the foundations of geometry and formal theories of arithmetic.

[CR46] Kreiser L (2001). Gottlob Frege: Leben - Werk - Zeit.

[CR47] Lambert JH, Engel F, Stäckel P (1895). Theorie der Parallellinien. Theorie der Parallellinien von Euklid bis auf Gauss.

[CR48] Lorenat J (2015). Polemics in public: Poncelet, Gergonne, Plücker, and the duality controversy. Science in Context.

[CR49] Mancosu P (2015). Grundlagen, section 64: Frege’s discussion of definitions by abstraction in historical context. History and Philosophy of Logic.

[CR50] Nagel E (1939). The formation of modern conceptions of formal logic in the development of geometry. Osiris.

[CR51] Pasch M (1882). Vorlesungen über neuere geometrie.

[CR52] Peano G (1894). Sui fondamenti della geometria. Rivista di Matematica.

[CR53] Plücker J (1831). Analytisch-geometrische Entwicklungen.

[CR54] Poncelet J-V (1822). Traité des propriétés projectives des figures.

[CR55] Resnik M (1974). The Frege–Hilbert Controversy. Philosophy and Phenomenological Research.

[CR56] Reye, T. (1866). *Die Geometrie der Lage*. Hannover: Carl Rümpler, 2nd, 1877 edition.

[CR57] Ricketts T, Haaparanta J, Hintikka J (1986). Objectivity and objecthood: Frege’s metaphysics of judgement. Frege synthesized.

[CR58] Ricketts T (1997). Frege’s 1906 Foray into metalogic. Philosophical Topics.

[CR59] Schiemer G, Reck E (2013). Logic in the 1930s: Type theory and model theory. Bulletin of Symbolic Logic.

[CR60] Schiemer G, Zach R, Reck E (2017). Carnap’s early metatheory: Scope and limits. Synthese.

[CR61] Schlimm D (2010). Pasch’s philosophy of mathematics. Review of Symbolic Logic.

[CR62] Shipley J (2015). Frege on the foundation of geometry in intuition. Journal for the History of Analytical Philosophy.

[CR63] Specker E (1958). Dualität. Dialectica.

[CR64] Tappenden J (2000). Frege on axioms, indirect proofs, and independence arguments in geometry: Did Frege reject Independence arguments?. Notre Dame Journal of Formal Logic.

[CR65] Tappenden J, Beaney M, Reck E (2005). Metatheory and mathematical practice in Frege. Gottlob Frege: Critical assessment of leading philosophers.

[CR66] Tappenden J, Ferreiros J, Gray J (2006). The Riemannian background to Frege’s philosophy. The architecture of modern mathematics: Essays in history and philosophy.

[CR67] Van Heijenoort J (1967). Logic as calculus and logic as language. Synthese.

[CR68] Veblen O, Young JW (1910). Projective geometry.

[CR69] von Staudt GKC (1847). Geometrie der Lage.

[CR70] Webb J, Hintikka J (1995). Tracking contradictions in geometry: The idea of a model from Kant to Hilbert. From Dedekind to Gödel: Essays on the development of the foundations of mathematics.

[CR71] Wehmeier K (1997). Aspekte der Frege–Hilbert–Korrespondenz. History and Philosophy of Logic.

[CR72] Wilson M (1992). Frege: The royal road from geometry. Noûs.

[CR73] Wilson M, Potter M, Ricketts T (2010). Frege’s mathematical setting. The Cambridge companion to Frege.

